# Tangata whaiora/consumers perspectives on current psychiatric classification systems

**DOI:** 10.1186/1752-4458-2-7

**Published:** 2008-06-12

**Authors:** Tess Moeke-Maxwell, Debra Wells, Graham W Mellsop

**Affiliations:** 1 Te Maia Cultural Services Ltd, Wellington, New Zealand; 2 Wellsprings Unlimited Trust, Hamilton, New Zealand; 3 Waikato Clinical School, University of Auckland, Hamilton, New Zealand

## Abstract

**Background:**

A number of studies have been undertaken with the aim of considering the utility of mental health classification systems from the perspective of a variety of stakeholders. There is a lack of research on how useful consumers/tangata whaiora think these are in assisting them in their recovery.

**Methods:**

Seventy service users were involved in seven focus groups in order to consider this question.

**Results and discussion:**

While for clinicians diagnosing someone might be a discrete event and easily forgotten as a moment in a busy schedule, most people in this study remembered the occasion and aftermath very clearly. The overall consensus was that whether being 'diagnosed' was helpful or not, in large part, depended on how the process happened and what resulted from being 'labeled' in the person's life.

**Conclusion:**

Overall, people thought that in terms of their recovery, the classification systems were tools and their utility depended on how they were used. They suggested that whatever tool was used it needed to help them make sense of their distress and provide them with a variety of supports, not just medication, to assist them to live lives that were meaningful to them.

## Background

The two most widely used mental health classification systems are those produced by the World Health Organisation (ICD-10)[1] and the American Psychiatric Association (DSM-IV-TR)[2]. A number of research projects have been conducted in New Zealand to ascertain the views of stakeholders on the usefulness of these classification systems [3-5]. The stakeholders included General Practitioners, mental health nurses, psychiatrists and clinical psychologists as they are most likely to be utilising a diagnostic or classificatory system to inform their assessments, diagnosis and ongoing treatment plans.

Given that there is a move towards tangata whaiora (preferred term for consumers in New Zealand from a consumer perspective), clinicians and mental health services developing recovery frameworks which emphasise consumer autonomy and participation in their recovery processes this project is unique in that it has sought the views of consumers. They are positioned as one of the knowledgeable stakeholders regarding the utility of classification systems, diagnostic process and interactions with clinical staff as they are in fact the ultimate stakeholder [6-10]. The purpose of this paper is to present consumer views on the utility of these systems in supporting them to move beyond the distress and circumstances of their presentation to recovery, that is living full and meaningful lives[8].

## Methods

Unlike the other stakeholders in the broader project, consumers do not generally have any in-depth knowledge about either the DSM or the ICD. It was therefore important to use a qualitative research methodology to access their experiential knowledge about the diagnostic process and the classification systems. This was achieved through seven focus groups which were digitally recorded and later transcribed. Focus groups were considered the most appropriate methodology as they provided participants with a forum to focus on the research themes through contributing to a guided discussion. This involved participants speaking and listening about their in-depth experiences, perceptions and personal narratives about the diagnostic classification system. This method helped to build a rapport and a sense of safety between participants and between consumers and the interviewers which increased the likelihood of in-depth communication and information gathering. The data derives its reliability from the quality and richness of the experiential data in terms of the subtleties, nuances and meanings that participants attributed to the issues they identified in the focus groups. The validity of the data is measured in terms of the value of participants' expertise based on their perceptions and experiential knowledge of the diagnostic classification system from a 'consumer' standpoint (Davidson and Tolich, 1999).

The findings presented in this report have been driven by the thematically coded narrative data utilising NVIVO software. The transcripts, facilitators' field notes and written material submitted by the participants also informed the data analysis [11]. The raw data will be destroyed 12 months after the study is published. The hard copy data will be stored in a locked cabinet and electronically protected on the researchers' personal computers.

The focus groups (two hours) were conducted in areas that reflected the cultural diversity and geographical spread of New Zealand consumers. The groups included one specifically Māori, one Pacific Nations' Peoples group and five culturally/ethnically random groups from the general population. Participants verbally agreed to maintain confidentiality at the start of each focus group. The focus groups and analysis were carried out by two consumers.

Approval for the project was obtained from the New Zealand Multiregional Ethics Committee.

### Participants

Seventy tangata whaiora participated in the focus groups with an average of 10 people per group; 32 men and 38 women participated. Their ages ranged from 26 – 68 years (mean 43.7 years). Of the total, 43 identified themselves as European/Pakeha, 11 as Māori and 7 as Māori/Pakeha. One person identified themselves as Australian, 4 as Samoan and 2 as Cook Island Māori. Two people did not record their ethnicity. In terms of service utilisation, 52 people had used both inpatient and community services and 10 had utilised community based services only. A large number of participants (56) identified their first diagnostician as a psychiatrist, two as a General Practitioner and two as mental health nurse. One person self-identified as their own diagnostician and four people could not remember who diagnosed them.

The range and number of current diagnoses included Schizophrenia (12), Bi-Polar Disorder (26) and Depression (17). Schizo-Affective Disorder (6), Post-Natal Depression/Psychosis (6) Post Traumatic Stress Disorder (1) and Borderline Personality Disorder (4). Three elected not to disclose this information.

## Results and discussion

### Pre-diagnosis

Participants variously spoke of four major factors which they thought impacted on their psychological state prior to diagnosis. These were their life experiences and social circumstances, their physical health, a family history of mental illness and their culture.

Many participants believed that their life experiences and social issues contributed to or caused their mental health problems

Many who had family members with a mental illness found it difficult to seek help due to having witnessed the treatment, stigma and discrimination that their relative had experienced, both within the family and in society at large. Some withheld how they were feeling from their families to protect them. One participant in this position said

*Well I had very little faith... no faith that people and professionals were able to help me upon receiving a diagnosis. I was very scared for my new born baby and I didn't believe I could get help... I was very scared what the drugs might do; I didn't have enough information*.

The culture of the participant also impacted on how they managed the experiences they were having. It was not uncommon for Māori to conceal their illness for a long period of time from family and friends before accessing support

Fearful, consumers withdrew from *whānau *(Family) and friends, those who would normally provide them with *aroha *(caring, kindness, love, compassion) and *manaakitanga *(support, reciprocal sharing and caring).

A similar scenario also existed for Pacific Island Nations' people. Pacific Island Nations consumers spoke about a culturally specific form of stigma and discrimination evidenced in their languages which describes people with a mental health problem as "broken head", "silly head" and "stupid". Indigenous phrases are powerful and induce shame; two of the phrases translate as "Your head is sick beyond the point of repair" and "Your brains are busted." Pacific Island Nations' participants stated their mental health problems were often interpreted as "makutu" or curses and families may speculate on the generational origins and contemporary implications of the mental health problem which is unhelpful. One participant said "There is a huge association with curses [families] might say "Oh, that girl has got a stupid head because her ancestor... did something wrong and this is what they get."

In fact, the tension created by an internalised culturally specific stigma prevented many Pacific Island Nations participants from informing their family members about the problem which, in turn, prevented them receiving professional support. Those who had family members with mental health problems were, again, less likely to access support and were often afraid.

Overall most participants in this study reported feeling scared, ashamed, lonely and confused prior to accessing help.

### Diagnostic assessment

While for clinicians the process of diagnosing someone may be seen as a discrete event, consumers experienced this process in terms of how it impacted on their sense of identity, sense of hope and their futures. Most of them clearly remembered receiving their diagnosis and how they felt about it at the time. Their initial responses fell into two categories; 'Relief and hope' or 'Confusion, disappointment and disillusionment'. Some, who initially felt relief, moved into the second category over time.

#### Relief and hope

The expectations of tangata whaiora were often high. Many commented that they felt a sense of relief as they believed their health would be fully restored to its former state. A participant stated:

*I was only 18. It was pretty tough because I was right out of school and had just finished my bursary – I mean I wasn't able to sit my exams because I had been ill with stress and insomnia. But the relief of me having that diagnosis... although it was traumatic and overwhelming at the time*.

Having received their diagnosis, a significant number felt "relieved" and "happy". Their problems were validated and their experiences normalised as they were "not the only one". A typical response in this regard was:

Initially it was a relief. The first one or two days were fantastic. It was like Yes, I know what is wrong with me. Now I might be able to fix it. So for me then I was like a dog with a bone and I went to the library and I read up everything I could on bi-polar...

Like others, Māori *tangata whaiora *experienced a sense of relief following their diagnosis. But Māori consumers often felt too *whakama *(embarrassed/ashamed) to discuss their assessment or treatment plans over time. Pacific Island Nations participants found it both challenging and confusing due in large part to language problems. They experienced a sense of relief as they "handed the problem over to the doctor". Some participants stated however that relief often turned to disappointment, disillusionment and anger when they did not recover as expected. In other words a diagnosis did not lead to a 'cure'.

#### Confusion, disappointment and disillusionment

While many participants felt relief, others commented they felt shocked, angry, confused or uncomfortable when they received their diagnosis. One person stated:

I came into contact with my first psychiatrist (that I could understand) who started throwing around words like "schizophrenia" and then "manic depression". Those words I instantly rejected because they meant "Crazy" [and] "Mad" and all those other things that I associated... particularly with schizophrenia. So there was total instant rejection; that is not something I could handle at all. So it wasn't a relief. It was just like "No, absolutely not!"

Some Māori consumers wondered why it took such a long time to receive a diagnosis.

A few Māori felt "devastated" as they were seeking support to help them manage their social issues and did not feel that receiving a diagnosis contributed to this. The Pacific Island Nations participants wondered how a diagnosis would benefit them. No explanations were given for how a diagnosis was made. Receiving a diagnosis also contributed to their problems as many lacked the support to understand the internal and external stigma and discrimination they faced as consumers both within their own culture and the broader community. Many Pacific people were also disappointed and disheartened at the length of time it took to receive a diagnosis.

Receiving a diagnosis and medication were viewed as synonymous by most participants. One stated:

I have a vague idea that psychiatrists would give me the medication that would help me function and that didn't [happen]. I thought that was automatic... logically part of the diagnosis and that they would help you get over it because you know that is what they do.... The reality is that it took around twenty five years for me to find any kind of medication that worked – twenty five years of my life!

Some had mixed feelings about receiving medication. Others commented on the difficulty of the process given that a variety of medication was often prescribed before a suitable one was found. For example, someone stated "I was trialed on three or four different anti-depressants and my skin reacted to most of them..."

The participants became disillusioned and angry when the medication failed to restore their former health and some refused to take it. One person stated it took three years to find the correct medication which they described as "terrible years". They said:

*That was hard going through all those years, trying all these drugs. And it made you slurp and dribble and... fall over. They were terrible years... until a psychiatrist came and said "I'm going to try an old fashioned drug". And so until I got the medication that suited I was going from one place to the other... I wouldn't go out because... I'd dribble and fall over... they were terrible years*.

Another person stated that treatment is a "Medical regime which goes on and on for years." Side effects can also be difficult as this person highlighted:

*Cause I was trialed on three or four different anti-depressants and my skin reacted to most of them... just side effects. So I just basically got out all the medical books from the Readers Digest, they were pretty good. And then just looked up all this medication and the side effects of it were just horrendous, I just went off it. [I said] "I don't want this – I don't want these side effects" so I went off it*.

#### Mitigating factors

There were a number of mitigating factors that shaped the participants experience of receiving a diagnosis. These included: the level of support from family, whanau and friends, what sort of information was provided, the use (misuse) of clinician 'jargon', the culture of both the clinician and the participant, as well as social and environmental factors in the person's life and how well these were addressed by the clinicians they were working with.

#### Impact of receiving a variety of diagnoses

The majority of participants in this study had received more than one diagnosis which, for some, caused a loss of faith in the diagnostic system and a loss of confidence in and cynicism towards clinicians. One participant stated:

*They didn't quite know where to put me so I started with factitious disorder, post-traumatic stress syndrome disorder, depression, borderline personality disorder, and schizophrenia. So yeah, I have sort of done the rounds a bit*.

Participants also commented on the transient psychiatric workforce as new psychiatrists generally provided new diagnoses which resulted in new medication. A new psychiatrist was seen to be more likely to change an existing diagnosis which meant people had to cope with withdrawal symptoms from previous medications as well as deal with the side effects associated with new medication. Many people stated they had received six or more diagnoses. One person stated:

*You have a monthly appointment to go and see a psychiatrist. Your psychiatrist leaves town you get a new psychiatrist and the new psychiatrist wants to prove that he is a better psychiatrist that the last one. So the first thing he is going to do is change your meds and so if you are not terribly well empowered with yourself you wind up getting a new med instead of putting up a fight and saying "No, no, just leave me with this one, you just give me what I am getting" (if that's working). Every time [there's] a new doctor, every time a new diagnosis, and therefore different drugs. It comes to a point where you say "Oh I am not going to believe your diagnosis"... [E]very time a new medication then you are going to have new withdrawal symptoms*.

New diagnoses brought transitional changes which were difficult for participants as this person described "I got re-diagnosed at 40 as bi-polar and it literally tore my world apart. I lost my five children, I lost my home, and I lost everything." Māori participants, like others, commented that there were too may clinicians involved in their assessment process which required them to keep repeating their stories. Medications were often changed without consultation or explanation. People felt disillusioned, confused and frustrated by the process. One person said that there seemed to be "few identifiable gains" to having one's diagnosis changed.

## Improvements to the classification systems

Participants were asked how they thought classification systems could be improved. Their responses fell in to two categories, the classification system itself and the process of assessment and diagnosis.

The first problem people identified within the classification system was that the multiaxial axis aspect, which they believed promoted a perception of hierarchy, and by implication seriousness of illnesses. This was particularly relevant to people who have been diagnosed as having Borderline Personality Disorder who felt that having an Axis 2 rather than Axis 1 diagnosis created huge difficulties for them in receiving appropriate treatment.

It was suggested that the numerical axis system should be abandoned and replaced with an alphabetical system.

A second issue that people discussed was the actual giving of a diagnosis. Participants discussed how the actual 'label' one received shaped their and others' perceptions and responses to them in a way that generally was not helpful.

The suggested solution to this was grouping symptoms. This entailed providing consumers and relevant others with 'lists' of symptoms and working together with the psychiatrist to identify what was actually troubling the person. One person stated:

*I think that's a brilliant idea that you're given the information [about symptoms] and out of this information is a tick list [detailing] where you think your symptoms sit, but also extending on the education and following it through. It may be assigning support workers to help with the education and information during that time*.

It was viewed that by doing this more emphasis, and by implication treatment, would be directed to what was causing the person's distress.

A Māori participant stated when consumers are involved in the diagnostic process it supports the recovery journey

*You feel like you own it. If you feel like you own it, then you're going to accept it and you're going to remember it. You're going find out more about it if you feel like you own it*.

The idea of grouping symptoms and providing checklists would also, participants believed, facilitate better understanding about how a particular 'diagnosis' was reached. This process, from the participants' perspective, seemed to be mysterious with often no correlation to, or acknowledgement of what they were experiencing. It was also felt that focusing on and treating symptoms might eliminate some of the risks associated with stigma and discrimination which can be based on the diagnosis or 'label' itself. A key component of this idea was providing information in everyday language about both the symptoms and any medication that was required. Participants felt that a better understanding of what they were being asked to take, what symptoms it was intended to help with, and possible side effects was vital.

This in turn would mean that the consumer and their family would be better informed.

The other category participants spoke of was in terms of the process of diagnosis. One of the issues was the length of time it seemed to take for psychiatrists to provide a diagnosis. In the main this was perceived as too short, that is after a one to two hour interview, but a few commented that it seemed to take years. The idea of an exploratory period was discussed whereby more attention could be paid to what the consumer and their family thought was happening. The exploratory period would take into account the person's complete history including their physical, emotional, mental, spiritual, familial and social situation to develop a comprehensive understanding of the person and their circumstances. This idea, much like the one of grouping symptoms, was also seen as an opportunity to build a relationship with the treating clinician in a manner that might engender a higher level of trust, understanding and collaboration.

The participants generally concluded that the diagnostic classification system, which they generally understood to mean being given a diagnosis or 'label' had no real value to them in terms of their recovery, but some stated it may function as a useful research tool.

## Conclusion

The purpose of this paper was to present consumer views on the utility of mental health classification systems in supporting them to move beyond distress to "recovery". The conclusion is that these systems are 'tools' and the power and influence of them on peoples' lives is more about the person using it than the tool itself. Overall, participants in this study have echoed what has been said in other publications [7,8,12,13]. They want to be involved in the process of both assessment and treatment in a way that helps them to make meaning of what they are experiencing. They want good quality information about both the 'illness' and the medications. They want purposeful and collaborative relationships with treating clinicians. They want services and practitioners alike to be culturally sensitive. The structure, the detail, the style and mode of use of the diagnostic classificatory system are all relevant if classification systems are to be useful rather than harmful to outcome.

## Competing interests

None of the authors have any competing interests. GM is a member of the ICD-11 strategic planning group of WHO.

## Authors' contributions

This project was conceptualized and managed by GM.It was planned by all three authors, with T M-m and DW doing the field work and analyses. The paper was writen by all three authors.
